# Viral Interference with Functions of the Cellular Receptor Tyrosine Phosphatase CD45

**DOI:** 10.3390/v7031540

**Published:** 2015-03-23

**Authors:** Nadine Thiel, Jasmin Zischke, Endrit Elbasani, Penelope Kay-Fedorov, Martin Messerle

**Affiliations:** Department of Virology, Hannover Medical School, Carl-Neuberg-Strasse 1, 30625 Hannover, Germany; E-Mails: thiel.nadine@mh-hannover.de (N.T.); zischke.jasmin@mh-hannover.de (J.Z.); elbasani.endrit@mh-hannover.de (E.E.); kay-jackson.penelope@mh-hannover.de (P.K-F.)

**Keywords:** CD45, viral immune modulation, evolution, positive selection

## Abstract

The receptor tyrosine phosphatase CD45 is expressed on the surface of almost all cells of hematopoietic origin. CD45 functions are central to the development of T cells and determine the threshold at which T and B lymphocytes can become activated. Given this pivotal role of CD45 in the immune system, it is probably not surprising that viruses interfere with the activity of CD45 in lymphocytes to dampen the immune response and that they also utilize this molecule to accomplish their replication cycle. Here we report what is known about the interaction of viral proteins with CD45. Moreover, we debate putative interactions of viruses with CD45 in myeloid cells and the resulting consequences—subjects that remain to be investigated. Finally, we summarize the evidence that pathogens were the driving force for the evolution of CD45.

## 1. Introduction

The receptor-like protein tyrosine phosphatase CD45 (also known as leukocyte common antigen (LCA) or protein tyrosine phosphatase receptor type C (PTPRC)) has been long acknowledged for its remarkably strong expression level on the surface of all nucleated cells of hematopoietic origin. In T cells it can make up to 10% of the cell surface proteins [1]. In immunological research, CD45 is therefore widely used as a marker for cells of myeloid and lymphoid lineage. CD45 is a typical type I transmembrane protein; it has two intracellular phosphatase domains and although only domain D1 is enzymatically active, both domains are required for optimal phosphatase activity [2]. A single transmembrane domain connects the intracellular part with the ectodomain, which consists of a cysteine-rich domain followed by three fibronectin III-like repeats. Due to alternative splicing of exons 4 to 6 of the CD45 gene, one or more of the three domains A to C can be added to the amino-terminal region of CD45, leading to the expression of different isoforms [3]. Although potentially eight CD45 isoforms could be formed by variations in splicing, only five have been found in nature (RABC, RBC, RB, RAB, R0) [4]. The domains A to C are O-glycosylated [5] and the cysteine-rich domain is highly N-glycosylated [6]. Depending on the isoform and the glycosylation state, the molecular masses of the CD45 isoforms reach 180–240 kDa. CD45 isoform expression and glycosylation are dependent on cell type and also on cellular maturation and activation state. These differences in cell surface-expressed CD45 may have an influence on its distribution in the plasma membrane and interactions with other proteins [7].

For many years, the function of CD45 was predominantly investigated in T lymphocytes and to a lesser extent also in B lymphocytes. Here we briefly summarize the essentials of CD45 functions, and for details we refer the reader to the many excellent reviews on the topic (for example references [8,9,10]). In T cells, one functionally important substrate of CD45 is the Src family kinase Lck [11,12], which activates downstream signaling following triggering of the T cell receptor (TCR) [13,14]. Lck can be both positively and negatively regulated by tyrosine phosphorylation leading to hyperactivation of T cells and reduced TCR signaling, respectively. A phosphorylated tyrosine residue in the active site (Y394) increases the kinase activity of Lck, whereas phosphorylation of a tyrosine in the regulatory carboxy-terminal domain (Y505) favors the formation of a closed conformation, resulting in diminished activity [9,15,16,17,18]. Upon engagement of the TCR, active Lck phosphorylates the immunoreceptor tyrosine-based activation motifs (ITAM) of the CD3 ζ-chain, which initiates a signaling cascade, resulting in the phosphorylation of downstream signaling proteins, such as ZAP-70, LAT and SPL76. Ultimately, this can induce cytokine production, differentiation and proliferation of T cells [19]. CD45 and the Csk kinase have opposing effects on the phosphorylation state of the negative regulatory tyrosine Y505, CD45 acts to dephosphorylate Y505, thus maintaining Lck in a “primed” state. The balance of the activities of CD45 and Csk determines the threshold at which a T cell can become activated [9,20,21]. Beautiful studies by the Vale group who reconstituted the TCR and the associated signaling network in a non-immune cell or in liposomes imply that in addition to Lck the ITAMs in the CD3 ζ-chain are a major target of the CD45 phosphatase and that spatial segregation of the TCR and of CD45 is the mechanism resulting in TCR phosphorylation [22,23]. Deletion of the CD45 gene disrupts signaling via the TCR and CD45-deficient mice develop severe-combined immunodeficiency (SCID) with defects in the development and function of lymphocytes [24,25,26]. Similar phenotypes have also been observed in patients carrying defective CD45 alleles [27,28,29].

In contrast to T cells, the development of Natural Killer (NK) cells is not disrupted in CD45-deficient mice, rather an increased number of NK cells was observed [30,31]. Although NK cells differ from lymphocytes of the adaptive immune system in that their receptors used for detection of molecular patterns of pathogens are not rearranged during development, the downstream pathways for transduction of activating signals in these different immune cells have many features in common, including the utilization of Src kinases and their regulation by CD45. Many NK cell-activating receptors signal via the adapter molecules DAP12, CD3ζ and FcεRIγ, which carry an ITAM motif. Upon receptor ligation the ITAM is phosphorylated by a Src family kinase, followed by the recruitment of one of the two Syk family kinases expressed in NK cells, Syk and ZAP-70. Those functions of NK cells that are induced by engagement of receptors associated with ITAM-containing adapters are either completely abrogated or strongly affected in the absence of CD45. This applies to cytokine production, cytotoxicity and proliferation of NK cells in response to viral infection [31,32,33,34]. Since Syk function is partially independent of Src kinase activity, cell-mediated cytotoxicity is still detectable in CD45-deficient NK cells, although at a reduced level. Cytokine (IFN-γ) production by NK cells in response to stimulation with pro-inflammatory cytokines (IL2, IL12, IL18) is CD45-independent [31], as this pathway employs different receptors and adapter molecules. Although not all NK cell functions have an absolute requirement for CD45, a recent study demonstrated that those functions remaining active in CD45-deficient NK cells were not sufficient to protect mice against an infection with mouse cytomegalovirus (CMV) [34], underlining the vulnerability of these innate effector cells to viral interference via CD45.

## 2. Viral Interference with CD45 Affecting the Activity of Immune Cells

Almost all viruses have to modulate the immune response—at least to some extent—to gain a foothold in their hosts. Immunomodulatory strategies, also termed viral immune evasion, seem to be particularly important for viruses that establish long-lasting persistent infections. Examples of viral immune evasion range from interference with the recognition of molecular patterns of viruses by cellular receptors of the innate immune system to modulation of effector mechanisms of the adaptive immune response.

A recent genome-wide mutagenesis screen in mice revealed that CD45 functions are essential for mounting a protective immune response against Herpes simplex virus type 1 (HSV-1) [35]. Since lymphocytes do not develop normally in the absence of CD45, this study underlines their pivotal role in controlling HSV-1 infection and in preventing HSV-1 associated encephalitis. Beyond this fact the study also suggests that CD45 represents a potential Achilles’ heel of the immune system that could be an attractive target for viral counteraction. Notably, excessive CD45 activity can also result in potentially lethal immune pathology, as observed during the infection of mice with Ebola virus infection [36] and with *Bacillus anthracis* [37]. It may therefore be not only to the benefit of the pathogen but also of the host to regulate CD45 activity.

CD45 regulation is a topic that has been actively discussed over decades by authors with many different viewpoints (for review, see reference [10]). The search for cellular ligands of CD45 has led only to the discovery of cellular interaction partners, such as lectins, whose binding to CD45 is mediated via glycans and is thus typically of low specificity. This does not, however, exclude physiologically relevant roles for such molecules in influencing CD45 activity as shown—for instance—by the modulation of effector T cell function caused by the macrophage galactose-type lectin expressed by immature antigen presenting cells [38].

Only recently, the first viral proteins that bind to CD45 have been identified [39,40]. Some members of the CMV RL11 gene family display hypervariability between different CMV strains, suggesting that they evolved under selection pressure and possibly interact with variable host proteins [41,42,43]. One of these hypervariable RL11 proteins is UL11, a predicted type I transmembrane protein [44]. Using a soluble fusion protein consisting of the UL11 ectodomain and the Fc-binding domain of human IgG, we detected that UL11 binds to the surface of various leukocytes, including B and T lymphocytes [39]. The interaction partner of UL11 turned out to be CD45, and the interaction could be confirmed not only for the soluble UL11-Fc protein but also for the full-length UL11 transmembrane protein when expressed at the cell surface using an adenoviral vector. UL11 binds to different CD45 isoforms with comparable efficiencies, suggesting that the binding site is within a domain common to all isoforms, which is consistent with the observed blocking of the interaction with an antibody specific for the conserved region of CD45 [39]. Pretreatment of T cells with UL11-Fc reduced their proliferation capacity upon TCR stimulation. This was associated with reduced tyrosine phosphorylation of signaling proteins, suggesting that binding of UL11 reduces the phosphatase activity of CD45, which in turn affects the pool of active Lck molecules and thus the transduction of signals received by the TCR. Based on these observations we hypothesized that UL11 protects CMV-infected cells against the effector mechanisms of virus-specific T lymphocytes. We could confirm that UL11 is exposed on the surface of CMV-infected fibroblasts, although at relatively low level [45]. Unexpectedly, CMV-expressed UL11 had no effect on the activation of CMV-specific T lymphocytes when assessed by co-cultivation with CMV-infected cells. This leaves a number of questions for further investigation. It remains to be analyzed whether UL11 is expressed at higher levels in cell types other than fibroblasts or under physiologic conditions *in vivo*. Another possibility is that UL11 does not principally target mature effector T cells but rather has effects on the priming of naïve T cells, under conditions where the presented antigens are possibly only available in limiting amounts and the activation threshold may be considerably higher. Given that a substantial amount of UL11 seemed to reside in the endoplasmic reticulum of infected cells, it is also conceivable that UL11 rather interacts with CD45 present within CMV-infected cells than on neighboring cells. CD45-expressing myeloid cells such as macrophages and dendritic cells have been shown to be important target cells of CMV infection [46]. One of our aims is therefore to examine the potential impact of UL11 on the functions of CD45 in these cell types.

**Figure 1:**
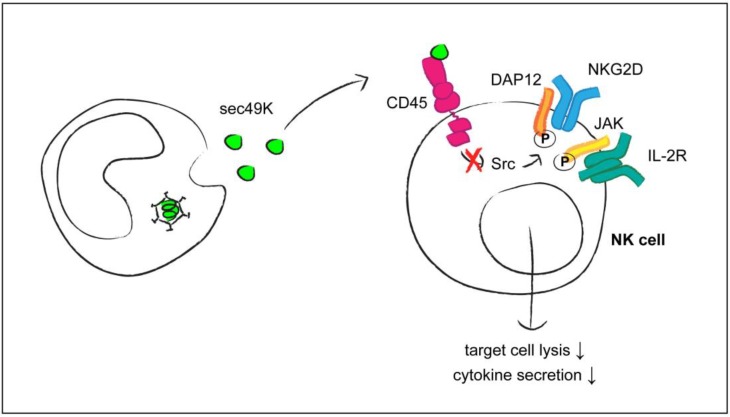
Modulation of NK cell functions by the secreted adenovirus type 19a sec49K protein (as described in reference [40]). Binding of sec49K to CD45 results in altered activity of Src kinases, affecting the phosphorylation of the adapter molecule DAP12 that is associated with the activating NK cell receptor NKG2D. CD45 can also regulate the phosphorylation of JAK kinases associated with the interleukin-2 receptor (IL-2R).

The second viral protein for which binding to CD45 has been reported is the E3/49K protein of adenovirus (AdV) type 19a, belonging to the AdV species D [40]. Interestingly, specific domains (CR1) within the N-terminal ectodomain of the E3/49K transmembrane protein [47] share some characteristics with a domain (RL11D) present in CMV RL11 proteins [41]. In contrast to the CMV UL11 protein, the Ad19a E3/49K protein is proteolytically cleaved, leading to the secretion of the ectodomain (sec49K). It is this soluble sec49K protein that binds to CD45 on the surface of T lymphocytes and NK cells as well as of B cells and monocytes [40]. Treatment of CD4 T cells with sec49K impaired their activation and led to a decrease in cytokine secretion upon stimulation with specific antigens. Functional impairment of T cells was associated with diminished phosphorylation of the signaling molecule ZAP-70 and of the downstream target molecules ERK-1/2. The strongest inhibitory effects of sec49K were seen for NK cells (Figure 1). Activation of these cells—induced by IL2 or by ligation of the NKG2D and 2B4 receptors—was reduced in the presence of sec49K, and NK cell-mediated lysis of MHC-negative target cells was strongly inhibited. The exact mechanisms of sec49K activity remain to be elucidated; it will be of interest to compare its effects in detail with those of CMV UL11 and of other lectin ligands for CD45. Altogether, this study points to NK cells as another population of immune cells that depend on CD45 function and that can be influenced by a viral protein.

One report has implicated CD45 in the apoptosis of CD4 T lymphocytes induced by the gp120 envelope protein of human immunodeficiency virus type 1 (HIV-1) [48]. Depletion of both infected and uninfected CD4 T cells occurs already early after infection in HIV-1 infected individuals and is later one of the hallmarks of progression to AIDS [49]. Engagement of CD45 has been linked with apoptosis, although the underlying mechanisms have not been completely elucidated and cell death may be due to triggering of unconventional apoptosis pathways (for review see References [50,51]). If CD45-mediated apoptosis contributes to gp120-dependent CD4 T cell depletion—in conjunction with several other mechanisms that have been suggested [52]—this would provide another example how a viral protein influences viral pathogenesis by means of CD45.

Polymorphism in the human CD45 gene—for instance the C77G and A138G mutations—change the expression patterns of CD45 isoforms and can result in an altered threshold for activation of T cells (reviewed in reference [53]). CD45 polymorphisms have been connected with altered susceptibility to autoimmune diseases and viral infections, for example with HIV-1 [54,55], hepatitis C virus [56] and hepatitis B virus [57]. Some of these studies may have been underpowered and have therefore been critically discussed and sometimes challenged; the association of CD45 polymorphisms with autoimmune diseases in particular has been disputed [58]. However, in view of the central role of CD45 in the immune response and in the control of viral infections there is a rational basis for this hypothesis, warranting larger epidemiological studies.

## 3. Utilization of CD45 to the Benefit of Viruses

In some circumstances viruses go beyond the functional inhibition of proteins involved in cellular defense mechanisms and even manage to integrate such molecules into their lifecycle in order to promote viral replication. One example that applies to CD45 is the human T cell leukemia virus type 1 (HTLV-1). Mazurov *et al.* (2012) detected that cell-to-cell transmission of HTLV-1 is inhibited by monoclonal antibodies which recognize a specific glycan signature (the so-called Tn antigen) attached to CD45 on Jurkat T cells (and also to another surface protein, CD43) [59]. Moreover, high levels of fully O-glycosylated CD45 and CD43 promoted HTLV-1 infection of neighboring cells. CD45 and CD43 are large rod-like proteins, which protrude from the cell surface and due to their large size, and the negative charge resulting from sialic acid present within their O-linked glycans favor repulsion of cells. Accordingly, CD45 has to be excluded from the immunological synapse [60,61] and perhaps also from virological synapses [62] in order to allow activation of immune cells and efficient cell-to-cell transmission of viruses, respectively. Based on the observed clustering of newly formed HTLV-1 virions on the surface of Jurkat T cells that express high levels of CD45 and CD43 and mechanisms of HTLV-1 transmission previously described by others [63], Mazurov *et al.* (2012) proposed that the high abundance of these proteins forces the virus to assemble only at specific sites of the outer membrane. These clusters might then serve as contact sites to the target cell, mediating efficient cell-to-cell spread. In cells expressing lower amounts of these proteins HTLV-1 budding occurs along the whole cell surface, which makes transmission less efficient. Although blocking of CD45 with antibodies promotes adhesion between neighboring cells, the authors argued that the inhibition of HTLV-1 infection observed in this case is due to exclusion of the virions from the contact sites [59]. It remains to be seen whether the proposed mechanism also plays a role in HTLV-1 infection of primary T cells. Interestingly, the authors also observed inhibition of HIV-1 transmission in Jurkat cells upon knock-down of CD45 expression [59]. However, the mechanism appeared to be different from the one described for HTLV-1 and details remain to be elucidated.

HTLV-1 does not seem to be the only virus that employs CD45-like proteins for infection. Upon inoculation into the arthropod vector *Aedes aegypti*, West Nile virus particles associate with the C-type lectin mosGCTL-1 present in the hemolymph of the mosquito [64]. The lectin-decorated virions are then recruited to a mosquito homolog of CD45 (mosPTP-1) on the surface of cells of various mosquito tissues, promoting attachment and viral entry. West Nile virus upregulates both the expression of the lectin in infected cells and also its secretion thereby facilitating further viral spread within the mosquito. It appears that evolution taught viruses at least twice to utilize CD45 or CD45-like molecules in such diverse hosts as humans and insects.

## 4. Further Possible Interaction of Viruses with CD45 in Myeloid Cells

CD45 is not only expressed by lymphoid cells, but also by myeloid cells, such as monocytes, macrophages and dendritic cells (DC). This is of interest as these cells are central to the initiation of immune responses and can be infected by some viruses, such as CMV, which utilizes them to modulate immunity, to disseminate within the organism [65] and to establish latency [66]. The state of knowledge of CD45 functions in these cells is, however, still limited in comparison to the profusion of publications describing the role of CD45 in lymphoid cells.

**Figure 2:**
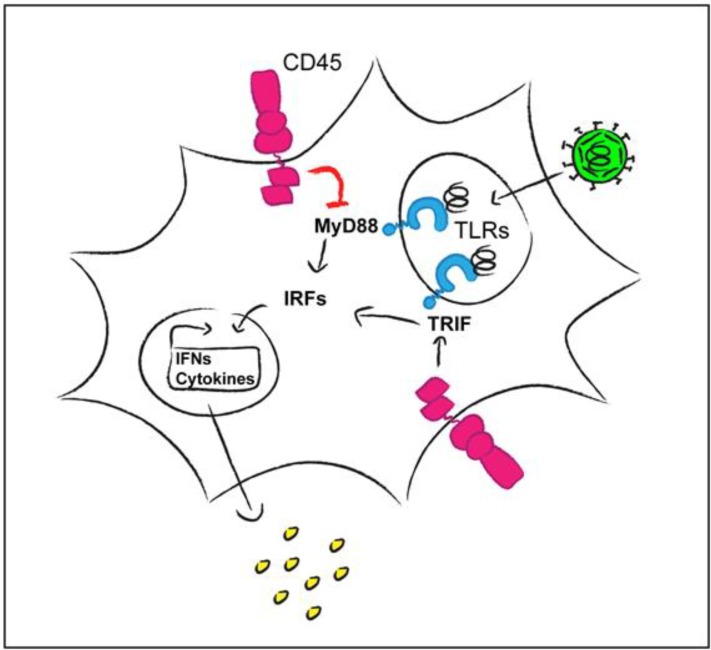
CD45 regulates TLR signaling in a positive or negative manner depending on the adapter molecules (MyD88 or TRIF) involved [68]. Viruses may modulate these pathways in order to influence cytokine production (IRFs, interferon response factors).

While T cells cannot develop normally in the absence of CD45, CD45 deletion has little impact on the development of DC with only a slight shift seen in the composition of DC subsets [67,68]. CD45-negative DC seem to be slightly pre-activated as assessed by the surface expression of co-stimulatory molecules. Piercy *et al.* (2006) observed that bone marrow-derived DC (BMDC) and splenic DC from CD45 knock-out mice responded with increased production of the pro-inflammatory cytokines TNFα and IL6 following stimulation with pattern recognition receptor TLR3 and TLR9 agonists [69]. In contrast, the levels of type I interferons (IFN) produced by plasmacytoid DC in the spleen upon infection with lymphocytic choriomeningitis virus were found to be diminished in CD45-/- mice [67]. These apparently contradictory outcomes in terms of the production of different cytokines could at least in part be explained by the results of another study [68], where it was shown that CD45 has a negative regulatory effect on TLR signaling pathways that depend on the adapter protein MyD88 and a positive regulatory role on MyD88-independent signaling (Figure 2). Mechanisms that were discussed [69] include the direct dephosphorylation of an adapter molecule by CD45 or—more indirectly—dephosphorylation of the negatively regulatory tyrosine residue of Src kinases, resulting in their activation. An involvement of Src kinases in TLR signaling has indeed been suggested [70]. Piercy *et al.* (2006) also found that cytokine production following stimulation with TNFα or IFNα was enhanced in CD45-negative BMDC. CD45 can apparently modulate the positive feedback loop of cytokine production and dampen the signaling induced by cytokine receptors. This is supported by the discovery that CD45 can dephosphorylate JAK kinases in various cells of hematopoietic origin, thereby negatively regulating cytokine-induced signaling [71]. A recent publication has implicated the adapter protein DOK-1 in the negative regulation of JAK-STAT signaling by CD45 [72].

The induction of a cellular antiviral state by cytokines (e.g., by type I interferons) is an important defense mechanism against many viruses; in line with the mechanism outlined above, replication of an interferon-sensitive Coxsackievirus was more strongly inhibited by IFNα in CD45-negative cells and CD45-/- mice were found to be much more resistant to infection with the Coxsackievirus than their CD45-positive littermates [71]. However, other factors than the diminished regulation of JAK-STAT signaling may have contributed to this phenotype of CD45-/- mice as they also lack T cells which are known to mediate immunopathological tissue damage [73]. To sum up, CD45 obviously plays a complex role in the sensing of pathogens and the orchestration of the antiviral immune response by myeloid cells. These functions of CD45 may potentially be modulated by viral proteins, although this remains speculative and has to be the subject of further experimental investigation.

Modulation of cell adhesion by CD45 in macrophages [74] is another interesting subject in the context of viral infection. Altered adhesion has also been reported for CD45-negative T cells [75,76], and it seems possible that the underlying mechanisms are similar, as in both cell types integrin signaling is affected upon CD45 deletion. In macrophages CD45 was found to co-localize with ß2-integrin and Src kinases in focal contact sites. Roach *et al.* (1997) reported that CD45-negative macrophages adhere and spread more rapidly than normal macrophages when plated on plastic dishes, however the CD45-negative cells were unable to maintain this β2 integrin-dependent adherence as they detached after 48 h. Moreover, the CD45-negative macrophages displayed altered morphology. These changes were associated with hyperphosphorylation and hyperactivation of the Src kinases Hck and Lyn. Binding of macrophages to the extracellular matrix or to neighboring cells via integrins induces a signaling process (outside-in signaling), which is necessary for adhesion, migration and also for immunologic activation. Upon integrin clustering Src kinases are rapidly activated and phosphorylate and activate focal adhesion kinases (FAK) and FAK-binding proteins (for a short overview see reference [77]). Based on the observations that the phosphatase activity of CD45 is required for its function in cell adhesion [75] and that CD45 and Src kinases are intimately connected in TCR signaling, a role for CD45 upstream of Src kinases has been proposed. However, in this process CD45 acts rather as a negative regulator, limiting the activity of the Src kinases by dephosphorylating the autocatalytic site [74]. A recent study sheds some light on the molecular mechanism by which CD45 regulates macrophage adhesion [78]. Using bone marrow-derived macrophages, St-Pierre and Ostergaard (2013) confirmed the phenotypic characteristics of CD45-/- macrophages described by Roach *et al.* (1997), and also detected impaired mobility of these cells [78]. Interestingly, the level of paxillin was found to be strongly decreased in CD45-negative cells due to its degradation by calpain, probably in response to tyrosine-phosphorylation of paxillin by members of the focal adhesion kinase family (FAK or Pyk2). Paxillin acts as a scaffold for coordination of the signaling processes at sites of adhesion [77]. As outlined above, FAK kinases are themselves targets for phosphorylation by Src kinases, which are hyperactive in the absence of negative regulation by CD45. Given that macrophages can serve as vehicles for dissemination of viruses, which involves the release of macrophages from cellular tissues into the blood stream followed by their subsequent adherence in other organs, it could be postulated that regulation of CD45 by viral proteins is a mechanism to govern this process.

## 5. CD45 Evolution in the Light of Recently Identified Putative Viral Ligands

**Figure 3:**
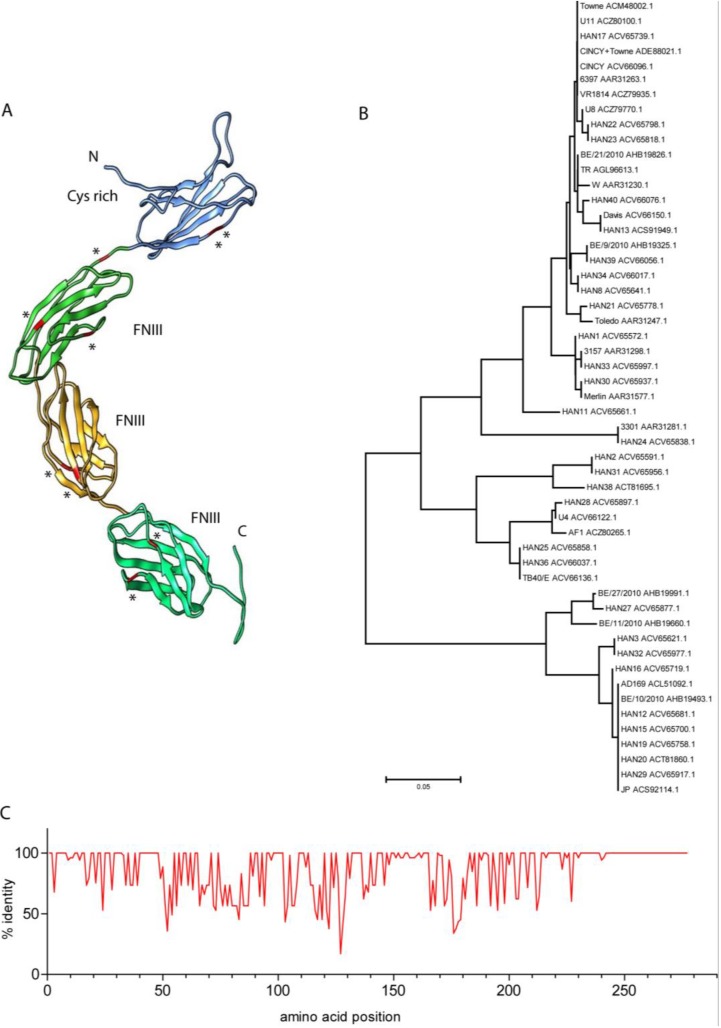
(**A**) The positions of those residues in the extracellular domain of CD45RO that were shown to be under positive selection [81] are labeled in red and marked with asterisks. The model of the CD45 structure was generated with the RaptorX program [84,85] and depicted using UCSF Chimera [86]. (**B**) Phylogenetic representation of 53 UL11 amino acid sequences derived from clinical and laboratory adapted HCMV strains. The phylogeny tree was calculated with MEGA 6 [87]. (**C**) The identity scores of the amino acids along the different positions of UL11 were calculated with the Jalview program [88].

Pathogen-host interactions are often marked by signatures of positive selection on host genes encoding the interacting proteins. These signatures are characterized by increased rates of non-synonymous (Ka) as opposed to synonymous (Ks) nucleotide substitutions, resulting in amino acid differences in the encoded proteins. The evolution of CD45 has been the focus of several studies [79,80,81], which revealed that the CD45 extracellular domain has been evolving under strong positive selection, whereas the intracellular domain is well conserved. An evolutionary study of primate genes potentially involved in viral pathogenesis identified ten residues in CD45 that were under positive selection [81]. Interestingly, all of these hot spots were located in the extracellular domain of CD45, mostly at the surface of the predicted structure (Figure 3A) and thus potentially accessible to contact by interacting proteins. Filip *et al.* (2004) compared the Ka/Ks rates in the alternatively spliced exons and two common exons encoding the cysteine-rich and fibronectin III-like domains of CD45 in primates. Alternatively spliced exons could be expected to evolve faster than constitutively spliced ones as the encoded protein domains may have more freedom to diverge, but the reverse was observed. The common exons displayed even higher rates of nucleotide substitutions than an intron of the primate CD45 genes (intron 6), although the latter would plausibly be able to tolerate a greater number of nucleotide changes. In both primate studies [80,81] it was hypothesized that the ectodomain of CD45 is targeted by pathogens—although at that time no pathogen was known to encode a CD45 interacting protein. The fact that the amino acid changes were mainly found in domains common to all CD45 isoforms suggests that pathogens may need to modulate the activity of all of the different CD45 molecules.

Notably, the HCMV UL11 and the AdV 19a E3/49K proteins bind to the constant region of CD45 [39,40], making these viral proteins candidates to have driven CD45 evolution. In a virus-host arms race model, one would not only expect signatures of positive selection in the host gene but also in the corresponding virus gene, particularly in the sequences encoding the part of the viral protein that mediates the interaction. The few studies that exist on CMV UL11 primarily revealed the highly polymorphic characteristics of the viral protein [42,44]. Alignment of the approximately 50 UL11 amino acid sequences available in the NCBI database and construction of a phylogeny confirms the reported divergence (Figure 3B). Interestingly, the polymorphism in the UL11 sequences is particularly pronounced at certain positions of the predicted extracellular domain (Figure 3C), whereas other parts of the UL11 protein, e.g., the signal peptide, the threonine-rich domain, the membrane-proximal part of the ectodomain and also the transmembrane region and the cytoplasmic tail are comparably well-conserved. It would be interesting to learn whether the divergent amino acids form part of the contact zone with CD45 and whether UL11 variants display different capacity for interaction with CD45. The few UL11 proteins which we tested were all able to bind to CD45 [39]; however, more careful evaluation would be needed to determine potential quantitative differences. Another interesting question is whether CMV UL11 proteins can bind to CD45 variants from primates, especially since the development of the RL11 gene family dates back to CMVs of primates [82]. Possibly, orthologous RL11 proteins of those primates CMVs exist that also bind to CD45 proteins. Since the high sequence variability of the RL11 proteins impedes the simple identification of UL11 orthologs, experimental testing of this hypothesis is required.

The apparently higher divergence of UL11 sequences compared to that of CD45 is in agreement with the predictions of a virus-host arms race as a virus has a much shorter generation time than its host, resulting in higher mutation rates. In addition, a nonstructural viral protein that is not absolutely required for virus replication and assembly has probably much more freedom to diverge than a host protein with importance for the immune response. In addition, abundant and broad expression in different cell types as is seen for CD45 can be expected to negatively affect the selection rate [83].

E3/49K-like proteins are apparently expressed by all AdV species of subgenus D [89], not just those causing epidemic keratoconjunctivitis as initially proposed. Whether all of the homologs of the AdV type 19a E3/49K protein share the ability of CD45 binding remains to be examined. Sequencing of whole genomes of species D human AdV revealed one of the highest amino acid variability in the predicted 49K (CR1-β) homologs among the AdV proteins [90]. Interestingly, homologous recombination between E3 genes of human AdV seems to be a driving force generating this diversity [90,91]. Functional analysis of these additional AdV E3/49K proteins and perhaps the identification of proteins from other viruses with similar properties may strengthen the support for the long-standing hypothesis that pathogens were the driving force behind the evolution of CD45.

## 6. Conclusions and Further Directions

A series of publications have reported on the interference of viruses from a range of different virus families with the functions of the receptor tyrosine phosphatase CD45. In view of the important and essential position of CD45 in various signaling networks of immune cells, it will not be surprising if more viruses and viral proteins are detected that take over command of these networks by either recruiting CD45 or modulating its functions. With the CMV UL11 and the AdV E3/49K proteins, now two proteins are known that can clearly bind to CD45. AdV E3/49K might be the first secreted viral protein identified that acts on CD45 in non-infected neighboring cells, possibly also on cells distant from infected tissues [40]. The precise function of the CMV UL11 protein is less clear; we hypothesize that it rather binds to CD45 in *cis* within CMV-infected cells. This is particularly interesting since CMV infects myeloid cells for which several specific functions of CD45 have been reported. However, the consequences of this interaction remain to be elucidated. For both the CMV UL11 and the AdV E3/49K protein the mode of binding and the resulting molecular effects for CD45 still have to be determined [92]. Both UL11 and E3/49K carry O- and N-linked carbohydrates and it is not clear whether and how these glycans contribute to the interaction. It is possible that the binding strength of these viral proteins is relatively weak and is mediated via the many glycans present in the extracellular domain of CD45, resembling the known interaction with several cellular lectins [93]. Engagement of CD45 by the viral proteins definitely has consequences for downstream pathways, but this could arguably be due to either increased or diminished phosphatase activity. The mechanism by which the activity of CD45 is regulated has been debated for many years. Rather than a conformational change of CD45, induction of dimerization or changed spatial distribution within the plasma membrane have been discussed as regulatory mechanisms. Experiments to determine the mode of action of the viral CD45 interaction partners could shed light on this issue.

Targeting of CD45 isoforms with specific antibodies has been proposed as a strategy to selectively modulate immune responses, for instance to induce tolerance against transplanted organs and to prevent rejection [94]—although with limited success. It is quite possible that the results of the long evolutionary interplay between viruses and their hosts can teach us about therapeutic options to allow us to intervene in the delicate and sophisticated signaling networks of immune cells in a much smarter way than has previously been conceivable.
